# Cancer screening in people living with HIV

**DOI:** 10.1002/cam4.6585

**Published:** 2023-10-25

**Authors:** Mar Masiá, Ana Gutiérrez‐Ortiz de la Tabla, Félix Gutiérrez

**Affiliations:** ^1^ Infectious Diseases Division Hospital General Universitario de Elche Elche Spain; ^2^ Centro de Investigación en Red de Enfermedades Infecciosas (CIBERINFEC), Instituto de Salud Carlos III Madrid Spain; ^3^ Medical Oncology Department Gregorio Marañón University Hospital Madrid Spain; ^4^ Department of Clinical Medicine Miguel Hernández University San Juan de Alicante Spain

**Keywords:** non‐AIDS cancer, screening, screening performance, surveillance

## Abstract

**Background:**

Cancer is the leading cause of mortality in people living with HIV (PWH) and is expected to account for a growing fraction of deaths as PWH age.

**Methods:**

In this literature review, we have compiled the most recent developments in cancer screening and screening performance in PWH, which are currently primarily implemented in well‐resourced settings. This includes an assessment of the associated benefits, harms, and cost‐effectiveness. The article also addresses unmet needs and potential strategies for tailored screening in the HIV population.

**Findings:**

Incidence and mortality due to screenable cancer are higher in PWH than in the general population, and diagnosis is frequently made at younger ages and/or at more advanced stages, the latter amenable to improved screening. Adequate evidence on the benefits of screening is lacking for most cancers in the HIV population, in whom standard practice may be suboptimal. While cancer surveillance has helped reduce mortality in the general population, and interest in risk‐based strategies is growing, implementation of screening programs in the HIV care settings remains low.

**Interpretation:**

Given the devastating consequences of a late diagnosis, enhancing early detection of cancer is essential for improving patient outcomes. There is an urgent need to extend the investigation in cancer screening performance to PWH, evaluating whether personalized measures according to individual risk could result in higher efficiency and improve patient outcomes.

## 1 INTRODUCTION

Life expectancy in people living with HIV (PWH) has risen dramatically since the advent of combination antiretroviral therapy (ART) but still lags somewhat behind that of the general population.^1^ One reason is probably the large, persistent difference in the comorbidity burden between populations, with cancer playing a central role in PWH.^2^ The so‐called non‐AIDS‐defining cancers (NADC), currently the dominant malignancies,^3^ are now the leading cause of death among PWH.^4^ The incidence and prevalence of numerous malignancies are much higher in PWH than in the general population. Moreover, they can occur at younger ages, be diagnosed at later stages, and cause higher mortality.^5^, ^6^, ^7^, ^8^

Although cancer is a major factor contributing to decreased survival in PWH, and cancer surveillance has been shown to reduce mortality,^9^, ^10^ available data on cancer screening in this population are scarce and mostly limited to small observational studies. HIV society guidelines recommend cancer screening with the same methods used in the general population.^11^ Unfortunately, for some cancers with the highest excess incidence and/or mortality in PWH (e.g., lung cancer and hepatocellular carcinoma), standard screening may show suboptimal performance.^8^, ^12^ Given the devastating consequences of late diagnosis, enhancing early detection is paramount to improving patient outcomes.

This review discusses the recent developments in cancer screening, predominantly implemented in well‐resourced settings, with a focus on the HIV population; the benefits, harms, cost‐effectiveness, and adherence to screening; and the unmet needs and potential strategies for tailored surveillance in PWH.

## 2 SCREENING FOR LUNG CANCER

Lung tumors are the NADCs with the highest incidence and mortality.^13^ In a meta‐analysis of 38 studies, the overall standardized incidence ratio (SIR) for lung cancer in PWH versus the general population was 2.5 (95% confidence interval [CI] 1.9–3.52).^5^ In PWH, lung cancer occurs earlier in life, and younger age groups have shown the largest SIRs, although the highest absolute excess risk is observed in people aged over 60 years.^6^ Mortality associated with lung cancer is up to four times higher in PWH^5^ (Table 1).

**TABLE 1:** Epidemiology of non‐AIDS–defining cancers and screening performance in people living with HIV.

Cancer	SIR, SMR compared to the general population	Clinical differences with the general population	Performance of recommended tests and compliance with screening in PWH	Implications for management/unmet needs
Lung	SIR 13.48–2.4^5^, ^6^ SMR 3.95^5,70^	Younger age at presentation^6^	In retrospective analysis of the incident lung cancers, low sensitivity, worse in women^12^, ^14^ Very low implementation rates^22^	Redefinition of criteria for LDCT screening with reduction of smoking and age thresholds. Risk stratification using biomarkers. Consider specific criteria for women. Awareness campaigns on the need for cancer surveillance
Liver	SIR 2.92–5.54^5,71^ aMHR 1.24 (95% CI 1.02–1.52)^72^	Younger age at presentation^73^ More advanced stage at diagnosis^74^	Low sensitivity to detect early‐stage HCC^8^ Low compliance rates^32^	Development of algorithms to stratify risk plus alternative imaging tests like MRI in high‐risk groups. Lower ultrasound interval and/or screening in non‐cirrhotic advanced fibrosis remain to be explored in PWH.
Anal	SIR 19.1–28.3^5^ SMR 124 (95% CI 27.3–563)^5^	—	Most studies performed in PWH; reduced progression to anal cancer in a RCT.^37^ Very low compliance rates^61^	Development of algorithms using clinical and molecular markers for HSIL prediction and progression followed by HRA and treatment of bHSIL.
Cervix	RR 6.07 (95% CI 4.4–8.4)^44^ SIR 3.80 (95% CI 3.48–4.15)^75^ aMHR 1.95 (95% CI 1.2–3.17)^76^	Proportion of adenocarcinoma substantially lower in WWH^77^	Higher frequency VIA‐positive in WWH^78^. Superior sensitivity of hrHPV for CIN2^+^/CIN3^+^ than VIA or cytology and improved specificity with 8‐hrHPV in WWH^79^ Lower adherence to the cervical cancer screening program, 29–46% ^80,81^	Need for a more precise identification of higher‐risk patients who may benefit from more intensive surveillance and/or screening methods
Colorectal	SIR 0.97–1.1^5,82,83^ SIR 2.49–28.9 for rectal cancer^34^, ^52^ SMR 2.09–2.53^5,58,82^	Younger age at presentation^53^	No data on performance in PWH Inconsistent data about compliance rates compared to the general population^51,62,84,85^	Earlier initiation of surveillance, especially in MSM with previous AIDS in whom rectal cancer is more frequent than in the general population. Consider risk factors such low CD4 or CD4/CD8 for risk stratification^86,87^
Breast	SIR 0.56–0.91^5^, ^54^ aMHR 1.90^58^ aHR 1.43–2.45^88^	Younger age, more advanced stage at diagnosis^56^, ^57^	No data on performance in PWH Low screening rates^61^	Earlier initiation, more frequent screening, need to assess alternative tests Campaigns to increase screening
Prostate	SIR 0.76 (95% CI, 0.64–0.91; I^2^ 91.6%)^24^ IRR 0.93 (95% CI 0.86–1.01) adjusting for PSA^64^ aMHR 1.28 (0.92–1.78)^58^	Gleason grade may be greater^64^	In a small case–control study conducted in the EuroSIDA cohort, the cutoff used in the general population showed low sensitivity in PWH^68^ Lower screening rates in PWH^64^	High‐level evidence is needed for PSA testing. Consider alternative surveillance tests such as ultrasound or MRI

*Note*: 10.1158/1055‐9965.EPI‐16‐0964^70^; 10.1200/JCO.18.00885^71^; 10.1016/j.jhep.2007.06.010^72^; 10.1097/00002030‐200,411,190‐00009^73^; 10.1093/cid/civ654^74^; 10.1097/QAD.0000000000002962^75^; 10.1200/JCO.2016.67.9613^76^; 10.1016/j.eclinm.2022.101645^77^; 10.1371/journal.pone.0263920^78^; 10.1016/j.eclinm.2022.101645^79^; 10.1186/1471‐2334‐14‐256^80^; 10.1097/QAD.0000000000001881^81^; 10.1097/QAI.0000000000001433^82^; 10.1016/S0140‐6736(07)61050‐2^83^; 10.1186/s12913‐015‐0711‐9^84^; 10.1111/j.1572‐0241.2005.50038.x^85^; 10.1093/jnci/djac053^86^; 10.1158/1055‐9965.EPI‐11‐0777^87^; 10.1097/QAD.0000000000002810^88^.

Abbreviations: aHR, adjusted mortality hazard ratio; aMHR, adjusted mortality hazard ratio; HCC, hepatocellular carcinoma; HRA, high‐resolution anoscopy; hrHPV: high‐risk human papillomavirus; (b)HSIL, (biopsy‐proven)‐high‐grade squamous intraepithelial lesion; LDCT, low‐dose computed tomography; MRI, magnetic resonance imaging; MSM, men who have sex with men; PSA, prostate‐specific antigen; PWH, people living with HIV; RCT: randomized controlled trial; RR, risk ratio; SIR, standardized incidence ratio; SMR, standardized mortality ratio; VIA, visual inspection with acetic acid; WWH, women living with HIV.

### 2.1 Screening tests

To date, only low‐dose computed tomography (LDCT) has been shown to reduce lung cancer mortality in two large randomized controlled trials (RCTs) in the general population.^9^ In PWH, available evidence with LDCT screening comes from a few small, uncontrolled observational studies,^14^, ^15^, ^16^ with heterogeneous inclusion criteria and short follow‐up.

Table 2 shows lung cancer screening methods and the level of evidence supporting them.

**TABLE 2:** Performance characteristics of cancer screening tests.

Cancer	Imaging test	Biomarkers	Genetic tests	Cytology	Endoscopy
Lung	‐ LDCT: reduced LC mortality in the NLST (IRR 0.85; 95% CI, 0.75–0.96) and NELSON (IRR, 0.75, 95% CI, 0.61–0.90) RCTs^9^ ‐ X‐ray: no benefit on mortality in an RCT^70^ ‐ MRI: Lung‐RADS scoring comparable between MRI and LDCT in an observational study^71^.	‐ High‐specificity blood‐based autoantibody biomarker followed by CT (EarlyCDT‐Lung test): reduced incidence of late‐stage LC but no mortality benefit in an RCT^72^ ‐ Blood, sputum, breath, and urine biomarkers: currently insufficient accuracy and/or high‐level evidence^73^ ‐ Predictive models with MRS‐based serum metabolomics: F1 score = 0.628 for LC prediction in serum samples obtained within 5 years before^74^	‐ Serum micro‐RNA signature classifier, and miR‐test: high NPV alone for detection of LC, and high S and NPV for LC mortality^75^ ‐ Airway gene expression classifier “Percepta”: high NPV for LC in non‐diagnostic bronchoscopies^76^	Sputum cytology: no clear benefit on mortality in an RCT^77^	
Liver	‐ Ultrasound: S 45% for early‐stage diagnosis in a meta‐analysis of observational studies^76^. Limitations: low sensitivity, particularly in cirrhotic patients with obesity and NASH^28^; low reproducibility.^31^ ‐ CT did not improve early diagnosis of HCC in RCT in general population^79^ ‐ MRI improved performance of ultrasound in observational study^29^	‐ Protein biomarkers: AFP, suboptimal S and Sp when used alone, but increases S of ultrasound for early detection of HCC to 63%^78^. AFP‐L3, DCP: poor performance^30^ ‐ Algorithms: GALAD (gender, age, AFP‐L3, AFP, DCP): low S, higher Sp. Doylestown (log AFP, AP, ALT, age, gender): discordant results. HES (AFP, AFP change, ALT, platelets): poor performance^30^	‐ Genetic biomarkers: DNA methylation, EV, cfDNA, miRNAs, lncRNA, circRNA: better accuracy, currently in early phases of validation^80^ ‐ Algorithm mt‐HBT (3 methylation biomarkers AFP, sex): higher sensitivity than GALAD and AFP^81^		
Anus		‐ Low CD4 counts associated with HSIL progression to cancer^41^ ‐ Low CD4/CD8 rate linked with higher risk of anal cancer^82^	‐ hrHPV DNA higher sensitivity than cytology but lower specificity for HSIL detection^38^ ‐ HPV16 higher specificity, lower sensitivity to detect HSIL and persistent infection with lower probability of HSIL clearance^38^ ‐ E6/E7 mRNA better specificity than cytology/hrHPV, but lower sensitivity^38^ ‐ Methylation biomarkers: greater levels in anal swabs from MSM with bHSIL^83^; the panel ZNF582, ASCL1, and SST showed the best discrimination to predict progression from HSIL to cancer^42^	Anal cytology: sensitivity 81% (95% CI 72%–87%;) specificity 62.4% (95% CI 54%–70%) at ASC‐US threshold to detect AIN2^+^ or worse^38^ ‐ Dual staining for p16/Ki‐67 no advantages over cytology or hrHPV^38^	High‐resolution anoscopy (HRA) for detection and treatment of bHSIL: 57% reduction of progression to anal cancer^37^
Cervix			‐ hrHPV: nearly 50% reduction in cervical cancer mortality; higher S for CIN3^+^ detection than cytology in RCT^84^; higher rates of colposcopy; high prevalence of transient HPV infection in women under 30 years old. ‐ HPV E6/E7 mRNA: effective to reduce colposcopy referrals and highly specific^85^ ‐ Methylation levels of the CADM1, MAL, and MIR124‐2 genes: AUC 0.80 to predict CIN2/CIN2^+86^	Cervical cytology decreased incidence of cervical cancer in ecological data^87^ Cotest leads to higher colposcopy rates than cytology and higher false‐positive rates^84^	Screen and treat: discrepant results about risk of recurrence after direct visualization with acetic acid and lesion treatment in WWH compared to uninfected women^88^
Colon‐rectum	Computed tomographic colonography (CTC): S 86% (95% CI 78%–95%) for lesions ≥6 mm and 89% (95% CI 83%–96%) for lesions ≥10 mm; Sp 88% (95% CI 83%–95%) for lesions ≥6 mm and 94% (95% CI 89%–100%) for lesions ≥10 mm^89^	‐ Guaiac‐based fecal occult blood test, the only fecal test to reduce mortality in RCTs^89^ ‐ Fecal immunochemical test, higher S/lower Sp (89%/94–95%) than gFOBT^90^ ‐ Blood septin 9: pooled S 62%, Sp 90%^10^ ‐ Elevated relative risk with CD4^+^ <200 cells/μL or with low CD4/CD8 ratios^82,91^	‐ Multitarget stool DNA (mtsDNA): S/Sp 92/88%^92^ ‐ Methylated SEPT9 DNA plasma assay (mSEPT9): S/Sp 69%/92%; NPV 99.9%^93^		‐ Colonoscopy: pooled reduction 47% (95% CI 29%–60%) CRC incidence, and 68% (95% CI 55%–76%) mortality in meta‐analysis of observational studies^88^ ‐ Sigmoidoscopy: pooled reduction CRC incidence 21% (95% CI 17%–25%) and 20% (95% CI 12%–29%) mortality after 15 years in meta‐analysis of 4 RCTs^94^ ‐ Colon capsule: S 87% for polyps ≥9 mm^95^
Breast	‐ Mammography: reduction of mortality in several RCTs^96^ ‐ Ultrasound: higher detection rates, including cancer missed by mammography; comparable to mammography in dense breasts^97^ ‐ MRI: higher sensitivity than mammography and ultrasound for all breast cancers, and of value in high‐risk women and in certain subgroups of higher‐than‐average‐risk women. Lower sensitivity for low‐grade ductal CIS^98^ ‐ Digital tomosynthesis: better sensitivity in dense breasts, less false‐positives^99^				
Prostate	Multiparametric MRI for risk assessment and eventually MRI‐targeted biopsy was superior to standard transrectal ultrasound‐guided biopsy in men with prostate cancer suspicion in an RCT^100^	PSA: modest reduction in prostate cancer mortality (IRR 0.96, 95% CI 0.85–1.08; low certainty) in a meta‐analysis; no benefit on overall mortality; associated with biopsy and cancer treatment‐related complications^65^ Percent‐free PSA, prostate health index or 4 K score may increase specificity and reduce the number of unnecessary biopsies^101^	PCA3 (prostate cancer gene 3) may reduce repeated biopsies for high PSA levels^101^		

*Note*: 10.1001/jama.2011.1591^70^; 10.1007/s00330‐018‐5607‐8^71^; 10.1183/13993003.00670‐2020^72^; 10.1016/j.jtho.2018.11.023^73^; 10.1073/pnas.2110633118^74^; 10.1186/s12885‐018‐4024‐3^75^; 10.1158/1055‐9965.EPI‐20‐0865^76^; 10.1002/cncr.24545^77^; 10.1053/j.gastro.2018.01.064^78^; 10.1111/apt.12370^79^; 10.3389/fonc.2021.583714^80^; 10.1016/j.cgh.2021.08.010^81^; 10.1093/jnci/djac053^82^; 10.1038/s41598‐022‐07258‐5^83^; 10.1001/jama.2018.10400^84^; 10.1002/ijc.34120^85^; 10.1097/QAI.0000000000000744^86^; 10.1097/COC.0000000000000264^87^; 10.1097/COH.0000000000000336^88^; 10.1001/jama.2021.4417^89^; 10.1177/2050640616659998^90^; 10.1158/1055‐9965.EPI‐11‐0777^91^; 10.1056/NEJMoa1311194^92^; 10.1136/bmjgast‐2019‐000355^93^; 10.7326/M22‐0835^94^; 10.1111/codi.13965^95^; 10.7326/M15‐0969^96^; 10.1186/s12885‐020‐06992‐1^97^; 10.1016/j.rcl.2020.09.004^98^; doi: 10.1007/s12282‐016‐0699‐y^99^; 10.1056/NEJMoa1801993^100^; 10.1515/cclm‐2019‐0693^101^.

Abbreviations: AFP, alpha fetoprotein; ALT, alanine aminotransferase; AP, alkaline phosphatase; AUC, area under the receiver‐operating characteristic curve; bHSIL, high‐grade intraepithelial squamous lesion in anal biopsy; cfDNA, cell‐free DNA; CI, confidence interval; CIS: carcinoma in situ; circRNA, circular RNAs; CRC: colorectal cancer; CT, computed tomography; DCP: Des‐gamma‐carboxy prothrombin; EV, extracellular vehicles; HCC, hepatocellular cancer; HES, HCC early detection screening; hrHPV, high‐risk or oncogenic human papillomavirus; IRR, incidence rate ratio; lncRNA, long noncoding RNAs; LC, lung cancer; LDCT, low‐dose computed tomography; lncRNA, long noncoding RNA; miRNAs, microRNAs; MRI, magnetic resonance imaging; MRS, magnetic resonance spectroscopy; MSM, men who have sex with men; mt‐HBT, multitarget HCC blood test; NLST, National Lung Screening Trial; NPV, negative predictive value; PSA, prostate‐specific antigen; RCT, randomized controlled trial; RR, relative risk; S, sensitivity; Sp, specificity.

### 2.2 Screening recommendations and performance in PWH

In 2013, the US Preventive Services Task Force (USPSTF) first recommended annual LDCT for individuals at high‐risk of lung cancer, with criteria updated in 2021.^9^ In Europe, few countries have committed to implementing LDCT screening, although implementation programs are currently underway in several countries.^17^ As of 2021, the European AIDS Clinical Society (EACS) guidelines endorse screening with LDCT where local screening programs are available, based on 2021‐USPSTF criteria (Table 3).^11^

**TABLE 3:** Recommended surveillance interventions for cancer screening in people living with HIV, benefits, harms, and cost‐effectiveness.

Cancer	Recommended surveillance test/target population for surveillance	Evidence on benefits in the general population and PWH	Harms	Cost‐effectiveness
Lung	LDCT/50–80 year‐old smokers of ≥20 pack‐years, active or quit <15 years ago^9^, ^11^	Reduced lung cancer mortality in general population in 2 RCTs. May detect early‐stage lung cancer, according to observational studies in PWH^14^, ^18^	FP results^a^ leading to unnecessary tests and invasive procedures, overdiagnosis, incidental findings, radiation‐induced cancer, and increases in distress. In PWH, no higher FP rates and similar or lower risk of radiation‐induced cancers than in the general population^14,18,70^	In PWH, screening with LDCT fell in the *efficiency frontier* ^b^ if 45–77 years, ≥20 pack‐years, CD4 ≥ 500 cells/μL and 100% ART adherence^18^
Liver	Semi‐annual ultrasound ± AFP/cirrhotic C‐P A/B and C awaiting liver transplant; non‐cirrhotic HBV infected, all or when PAGE‐B score ≥ 10^11^ ^,71,72^	Reduced mortality in individuals with HBV infection in one RCT. In cirrhosis, improved early HCC detection, curative treatment, and increased survival in observational studies in the general population.^31,73^	In 3 studies, physical harm due to FP/indeterminate results occurred in 9%‐27.5%, ranging from mild (cross‐sectional image scans), to severe effects (liver biopsy or angiogram); mild intensity in most studies. No data on psychological and financial harms. No data in HIV^74‐76^	In cirrhosis, cost‐effective if incidence HCC >1.5% p‐y^77^. In compensated cirrhosis, ultrasound + AFP cost‐effective if HCC incidence >0.4% p‐y and adherence >19.5%^78^; for hepatitis B, if HCC risk >0.2% p‐y. No data with NAFLD; IR of HCC in NAFLD‐related cirrhosis of 3.78 per 100 person‐years (95% CI, 2.47–5.78; I^2^ = 93%) in a meta‐analysis^79^. MRI cost‐effective for annual HCC risk >3%.^33^ No studies in HIV
Anal	DARE ± (Cytology ±HR‐HPV) ± HRA every 1–3 years/≥35 years MSM; transgender women and in persons with HPV‐associated dysplasia^11^, ^36^	Reduced progression to anal cancer in an RCT in PWH^37^	Usually well tolerated. Anxiety related to false‐positive cytology and procedural discomfort from HRA and biopsy^80^. ANCHOR RCT: 0.1% adverse events, and 0.04% serious adverse events^37^	Old studies; cost‐effective in USA and non‐cost‐effective in UK studies^81^. Studies pending after results of the ANCHOR trial. Regular DARE was likely cost‐effective in MSM living with HIV >50 years old^82^.
Cervix	PAP smear or liquid based cervical cytology test 1–3 years/21 years^6^; PAP/Co‐testing every 3 years for ≥30 years.^48^ Colposcopy if ≥LSIL on PAP, ASCUS and hrHPV, HPV16, or confirmed hrHPV with normal PAP^48^	‐ Screening reduced mortality in an RCT (risk ratio 0.65, 95% CI 0.47, 0.90)^45^; and from 41% to 92% in observational studies^83^ ‐ Similar benefits with screening in WWH^50,84^	Treatment‐related adverse obstetric outcomes; CIN that would have regressed without treatment; physical discomfort; anxiety precipitated by FP findings. hrHPV tests show higher FP and colposcopy rates than cytology, which may induce potential harms^85^. No higher morbidity from screening in WWH^86^	A cost‐effectiveness analysis comparing cervical cytology, VIA and HPV DNA testing for detecting cervical HSIL in WWH in Johannesburg, South Africa showed that VIA was most cost‐effective^87^. Annual Pap after two negative smears obtained 6 months apart, most cost‐effective in WWH^88^
Colorectal	Colonoscopy every 10 years or annual fecal immunochemical testing or multitarget stool DNA every 3 years with a life expectancy >10 years/50–75 years^c^ ^,10,11^	Only the fecal gFOBT has been shown to reduce incidence and mortality of CRC in RCTs in general population.^10^ Colonoscopy reduces CRC incidence and mortality in observational study.^10^ No data in HIV	Complications of endoscopy: bleeding (pooled event rate of 8 per 10,000), perforation (pooled event rate of 4 in 10,000)^89^, electrolyte imbalance and nephropathy from bowel preparations, post‐colonoscopy CRC (1 per 3174 colonoscopies).^10^ No specific data in HIV	‐All screening methods cost‐effective compared with no screening; too much heterogeneity to compare methods^90^ ‐ Of innovative tests, mSEPT9 was the most cost‐effective but led to a high rate of colonoscopy referral^91^ ‐ Initiation at 45 years likely cost‐effective^92^ ‐ No data in PWH
Breast	Biennial mammography/women 50–74 years^11^, ^59^, ^60^	Mammography reduced mortality in RCTs in general population.^60^	False‐positive (more common with annual frequency, younger age, and dense breasts) and false‐negative results, overdiagnosis, anxiety, pain during procedures, and radiation exposure (2–11 radiation deaths per 100,000 women using mammography)^93^. No specific data in HIV	‐ Biennial screening at 50–69 years or 50–74 years, the most efficient.^59^, ^60^ ‐ Screening improved QALYs and was cost‐effective for women 40–64 years, with an incremental cost‐effectiveness ratio of USD 50,223/QALY versus no screening^94^ ‐ MRI in its current form would not be cost‐effective^95^. ‐ No data in WWH
Prostate	PSA every 1–2 years if shared decision‐making/>50 years with a life expectancy >10 years^11^, ^67^	Meta‐analysis from RCT: modest effect to reduce prostate cancer mortality (IRR 0.96, 0.85 to 1.08; low certainty) but did not have a benefit on overall mortality and was associated with biopsy and cancer treatment‐related complications.^65^ No specific data in HIV	‐FP results leading to additional diagnostic procedures or prostate biopsy, overdiagnosis and overtreatment.^67^ ‐ Potential treatment complications include incontinence and erectile dysfunction^67^. No specific data in HIV	Uncertain cost‐effectiveness in a systematic review of decision‐analytical models based on the heterogeneity to reflect cancer progression, the inconsistent use of Gleason grade, the absence of reference to clinical verification, overdiagnosis and overtreatment, or inadequate methods used to assess quality of life^96^ No data in PWH

*Note*: 10.1097/QAD.0000000000001600^70^; 10.1016/j.jhep.2018.03.019^71^; 10.1002/hep.29086^72^; 10.1007/s00432‐004‐0552‐0^73^; 10.1002/lt.25398^74^; 10.1002/hep.28895^75^; 10.1016/j.cgh.2020.09.014^76^; 10.1016/S0002‐9343(96)00197‐0^77^; 10.14309/ajg.0000000000000715^78^; 10.1016/j.cgh.2021.05.002^79^; 10.1007/s11904‐011‐0085‐5^80^; 10.1071/SH12017^81^; 10.7448/IAS.19.1.20514^82^; 10.1016/j.ejca.2019.12.013^83^; 10.1097/QAD.0000000000000163^84^; doi:10.1001/jama.2018.10400^85^; 10.1371/journal.pone.0263920^86^; 10.1097/COH.0000000000000336^87^; 10.7326/0003‐4819‐130‐2‐199,901,190‐00003^88^; 10.1001/jama.2021.4417^89^; 10.2147/RMHP.S262171^90^; 10.1093/jnci/djaa103^91^; 10.1053/j.gastro.2019.03.023^92^; 10.7326/L18‐0570^93^; 10.1007/s10552‐019‐01178‐y^94^; 10.1016/j.rcl.2020.09.004^95^; 10.1186/s12885‐017‐3974‐1^96^.

Abbreviations: AFP, alpha fetoprotein; ART, antiretroviral therapy; CI, confidence interval; CIN, cervical intraepithelial neoplasia; C‐P, Child‐Pugh score; CRC, colorectal cancer; DARE, digital ano‐rectal examination; FP, false positive; gFOBT, guaiac fecal occult blood test; HCC, hepatocellular cancer; IR, incidence rate; IRR, incidence rate ratio; HSIL, high‐grade squamous intraepithelial lesion; LDCT, low‐dose computed tomography; MRI, magnetic resonance imaging; MSM, men who have sex with men; NAFLD, non‐alcoholic fatty liver disease; QALY, quality‐adjusted life years; p‐y, per year; PAP, Papanicolau; PWH, people living with HIV; RCT, randomized clinical trial; RC, randomized controlled; VIA, visual inspection with ascetic acid; WWH, women with HIV.

^a^ Different measures have been implemented to reduce false‐positive results, such as volume‐based nodule‐management,^21^ or using the Lung‐RADS American College of Radiology's classification system.

^b^ Efficiency frontier refers to the relative efficiency of the new intervention with the incremental cost‐effectiveness ratios (ICERs) of non‐dominated comparators.

^c^ The USPSTF recommendations (2021) extend the age range for screening to encompass adults aged 45–49, with a grade B recommendation.

In a simulation model developed in the US cohort of veterans living with HIV, and with a CD4^+^ count ≥500 cells/μL and 100% ART adherence, the estimated potential reduction in cancer mortality was 18.9% with the 2013‐USPSTF criteria, similar to that of the general population.^18^ However, a retrospective analysis of the incident lung cancers diagnosed in the ANRS‐CO4 French Database HIV cohort showed that only 31% would have been detected using the 2013‐USPSTF criteria. Lowering the age and smoking thresholds would have increased sensitivity.^19^ In a similar study conducted in the MACS/WIHS cohorts, the 2021‐USPSTF criteria outperformed 2013‐USPSTF criteria in detecting lung cancer but still showed suboptimal sensitivity, particularly among women.^12^ The inclusion of HIV‐associated factors did not improve accuracy, but lowering the age threshold and decreasing the pack‐year history criterion increased sensitivity in both cohorts, particularly in women, suggesting sex differences in lung cancer risk that warrant further exploration in future prediction models.

Table 3 describes the harms and cost of the screening strategy recommended in the general population and in PWH.

### 2.3 Implications for management

Besides being the most frequent NADC, lung cancer is also projected to be the most common cancer type by 2030, when the proportion of PWH older than 65 years is expected to double.^20^ The benefits and cost‐effectiveness of LDCT might be even higher in PWH given the greater incidence of lung cancer. Contrary to initial hypotheses, the frequency of false‐positive results is not higher in PWH.^14^, ^16^ Additionally, different measures have been adopted to reduce false‐positives rates, like volume‐based nodule‐management, or the Lung‐RADS American College of Radiology's classification system.^9^, ^21^ However, the 2021‐USPSTF criteria should be adapted to PWH. Approaches such as lowering the thresholds for age and smoking exposure or using biomarkers to stratify risk warrant further investigation with RCTs, including differential criteria by sex. A small single‐LDCT study that reduced the age threshold to 45 years found a high prevalence of lung cancer in PWH.^15^ Another relevant aspect to tackle is the low rates of lung cancer screening referrals in PWH.^22^ Compared to the general population, PWH may face more frequent and distinct barriers to lung cancer screening, both at the provider and patient levels. One such obstacle is the higher burden of comorbidities, particularly in the aging PWH, which may add complexity to the implementation of the screening procedures. However, there are also factors that promote screening within this population, which may offset these barriers. These factors include more common healthcare engagement, enduring and close patient‐physician relationships, greater patient familiarity with evidence‐based practices and willingness to undergo preventive interventions in the context of their long‐term survival awareness.^23^

## 3 SCREENING FOR HEPATOCELLULAR CARCINOMA

Hepatocellular carcinoma (HCC) is a common cancer with an increasing incidence rate (IR) in PWH^24^, ^25^ and the second most common cause of NADC‐related death.^13^ HIV infection confers a higher risk of developing HCC and lower survival than the general population.^5^ Non‐alcoholic fatty liver disease (NAFLD), currently the fastest growing cause of HCC in Western countries, shows a higher prevalence in PWH.^26^ Since 2018, nonviral liver disease has surpassed hepatitis C virus (HCV) as the leading indication for liver transplant in PWH in the United States, and non‐alcoholic steatohepatitis (NASH) accounted for nearly half of nonviral causes.^26^

### 3.1 Screening tests

Ultrasound is the primary test for HCC screening. Observational studies have reported that ultrasounds are beneficial for early diagnosis and mortality reduction in people with viral cirrhosis.^27^ Sensitivity is particularly low in cirrhotic patients with obesity and NASH,^28^ two conditions expected to increase in the near future. Magnetic resonance imaging (MRI), but not computed tomography (CT), has shown higher HCC detection rates than ultrasound.^29^ Blood biomarkers and algorithms including biomarkers plus clinical data have shown suboptimal results, but more recent genetic testing has demonstrated improved accuracy to predict HCC.^30^ Performance data on HCC screening methods are limited in PWH.^8^

Most surveillance studies have been conducted in individuals with viral hepatitis, but the benefits and strategies of screening in patients with NAFLD‐related cirrhosis have yet to be elucidated.

Table 2 shows the screening methods for HCC detection.

### 3.2 Screening recommendations and performance in PWH

Professional society guidelines recommend screening for HCC in high‐risk individuals, with consensus for cirrhosis of any etiology and higher variability for other risk groups^31^ (Table 3). The screening modality of choice is semi‐annual ultrasound. No agreement exists regarding the addition of alpha fetoprotein,^31^ though this combination has been estimated to be more cost‐effective than ultrasound alone in people with compensated cirrhosis.^30^

Screening recommendations in PWH are in line with those of the general population.^11^ There is a paucity of data on the accuracy of ultrasound for diagnosing HCC in PWH. An observational study demonstrated lower performance in detecting early‐stage HCC compared to HIV‐negative individuals, with a rate of screening failure of 57% vs 29%, respectively, and lower survival rates.^8^ Compliance with hepatocellular carcinoma screening is low in PWH.^32^

Table 3 summarizes the harms and cost of the recommended screening strategy for HCC.

### 3.3 Implications for management

The poor performance observed with ultrasound will presumably be aggravated by the growing prevalence of NAFLD in PWH. Both awareness campaigns on the need for surveillance and research on alternative screening strategies are imperative. Shorter intervals between ultrasounds and/or screening in non‐cirrhotic advanced fibrosis remain to be explored in PWH. Using a Markov model, MRI was cost‐effective in a subgroup of HIV‐negative patients with compensated cirrhosis and annual HCC risk over 3%, identified via a scoring system.^33^ Similarly, HIV‐adapted screening testing, preceded by risk stratification to select the most suitable candidates, would contribute to increasing the efficiency of the process. New algorithms including clinical variables and blood biomarkers, particularly the promising genetic biomarkers, could be candidates to stratify HCC risk.

## 4 SCREENING FOR ANAL CANCER

Of all the NADCs, invasive anal cancer is the malignancy with the highest excess incidence and mortality.^5^, ^34^ Anal cancer represents the third NADC with the highest mortality after lung and liver cancer,^13^ and the first NADC in years of life lost in PWH in the United States^35^ (Table 1).

### 4.1 Screening tests

The natural history of anal cancer can be modified by early treatment of high‐grade squamous intraepithelial lesion (HSIL), the necessary precursor of invasive carcinoma, to prevent progression to anal cancer.^36^ Unlike other types of cancer, most information about anal cancer screening derives from the HIV population. Furthermore, the only RCT demonstrating the benefits of anal cancer screening has been conducted in PWH. Diagnosis and treatment of HSIL through high‐resolution anoscopy (HRA), a procedure adapted from cervical cancer screening, has been shown to prevent anal cancer in the Anal Cancer HSIL Outcomes Research (ANCHOR) study, published by Palefsky et al.^37^ This phase 3 trial included 4446 PWH aged 35 years or older with biopsy‐proven anal HSIL (bHSIL) who were randomized to treatment versus active monitoring without treatment of bHSILs through biannual HRA. The initial treatment was electrocautery ablation in 83.6% of participants, infrared coagulation in 4.8%, and ablation or excision under anesthesia, topical fluorouracil, and topical imiquimod in the remainder. After a median follow‐up of 25.8 months, there was a significant, 57% (95% CI 6% to 80%) reduction in the rate of progression to anal cancer in the treatment group. The frequency of adverse events was low, and two‐thirds of cancers were diagnosed at stage I or II.

Despite the proven benefits, HRA has currently limited availability due to the scarcity of expert capacity and infrastructure. The procedure should therefore be reserved for people at the highest risk of cancer. Accurate screening tests for selecting the individuals with the highest pre‐HRA probability of bHSIL are particularly important to optimize the procedure. Although there is still no consensus, the most widely used screening test for referral to HRA is anal cytology; however, performance is suboptimal.^38^ Molecular tests including oncogenic strains of human papillomavirus (hrHPV) DNA, hrHPV E6/E7 mRNA, and DNA methylation have shown advantages in sensitivity or specificity but have not outperformed cytology.^38^ Screening algorithms combining two or more tests merit further investigation. A recommendation for anal cancer screening in high‐risk PWH is currently undergoing evaluation by the USPSTF.

Available screening tests to predict bHSIL are shown in Table 2.

Although the prevalence of bHSIL is up to 47% in men who have sex with men (MSM) with HIV,^38^ only a small proportion progress to cancer^39^ and spontaneous regression has been described.^40^ Accordingly, another aspect to be considered in screening is the detection of the bHSILs with the highest probability of malignant transformation, and therefore candidates for ablative therapy. DNA methylation, anal condyloma, low CD4 counts, and CD4/CD8 rate have been linked with a higher risk of bHSIL progression and anal cancer^41^, ^42^ (Table 2).

If early treatment of HSIL through HRA is not available, digital anal rectal examination (DARE) may allow early diagnosis of invasive carcinoma. Data on the benefits of DARE for early diagnosis of anal cancer are extremely limited. The size of the tumor is an important prognostic factor, and in one phase II clinical trial, the procedure was able to detect tumors of 3 mm or more.^43^ The optimal time interval remains to be defined.

### 4.2 Screening recommendations and performance in PWH

To date, there is no international consensus on routine anal cancer screening. Due to the preponderance of anal cancer in PWH, some HIV management guidelines recommend screening with DARE, with or without anal cytology plus HRA if abnormal, usually in MSM/transgender women and in persons with HPV‐associated dysplasia,^11^, ^36^ but protocols differ between centers.

### 4.3 Implications for management

The relevance of the results of the ANCHOR trial will directly impact cancer screening guidelines. Anal cancer screening with HRA should be incorporated as a routine procedure among the screenable cancers in people at risk. This implies a need for training in HRA on a large‐scale, ideally to infectious disease specialists, who are the physicians with the greatest involvement in PWH care. There is also a need for standardized protocols to select patients for HRA. A crucial step to optimize the procedure is to stratify people according to their anal cancer risk in order to identify the best candidates both for screening and treatment. Algorithms combining clinical/biological risk factors with biomarkers for bHSIL prediction and for bHSIL progression might be helpful for personalized anal cancer screening, warranting further research.

## 5 SCREENING FOR CERVICAL CANCER

In women with HIV (WWH), the risk of developing cervical cancer is six‐fold that of the general population,^44^ probably due to higher rates of persistent HPV infection (Table 1).

### 5.1 Screening tests

Treatment of precancerous lesions (HSIL) can prevent the development of invasive carcinoma and reduce mortality. Multiple observational studies and at least one RCT have shown that different screening strategies—even if performed only once in a lifetime—decrease mortality, reduce incidence of invasive cancer, and increase cure rates.^45^ The available screening strategies are based on early detection of HSIL through cervical cytology, testing for hrHPV, and co‐testing with both methods. Patients with abnormal results are referred for colposcopy and directed biopsy, and subsequent local treatment of HSIL or follow‐up of lesions with lower risk. “Screen‐and‐treat” methods, which imply testing and treating a positive result at the same visit, are accepted in resource‐limited regions^46^, ^47^, ^48^ (Table 2).

### 5.2 Screening recommendations and performance in WWH

The American Society for Colposcopy and Cervical Pathology (ASCCP) 2019 guidelines provide specific screening recommendations for high‐risk groups such as WWH, and these recommendations have been adopted by other societies.^11^, ^47^, ^48^ Screening through cytology should begin within a year of first insertive sexual activity or by age 21 years (HPV testing is not recommended before age 30 years because of the high incidence of HPV in this age group). Screening should be on an annual basis for the first 3 years, and once every 3 years thereafter. After 30 years of age, cytology or co‐testing should be conducted every 3 years throughout a woman's lifetime (Table 3). In resource‐limited settings, WHO recommends HPV DNA detection in a screen, triage and treat approach every 3 to 5 years from age 25.^46^ Table 1 shows screening performance in WWH, whose adherence to screening recommendations is lower than in uninfected women.^49^

### 5.3 Implications for management

WWH are a high‐risk group, and guidelines recommend longer and more frequent screening than in the general population. The rate of false‐positives is high,^50^ highlighting the need for a more precise identification of high‐risk patients who may benefit from more intensive surveillance and/or from screening methods with greater specificity and positive predictive value to avoid unnecessarily invasive procedures.

## 6 SCREENING FOR COLORECTAL CANCER

The incidence of colorectal cancer (CRC) seems to be similar in PWH and the general population^5^, ^51^; however, two studies have reported a higher frequency of rectal carcinoma^34^ and specifically of rectal squamous cell carcinoma,^52^ presumably associated with HPV and immunosuppression. PWH have been diagnosed with CRC at younger ages^53^ and have shown higher CRC‐associated mortality.^5^

### 6.1 Screening tests

Screening can prevent CRC by diagnosing and treating premalignant adenomas or sessile serrated lesions in early stages through endoscopy. Screening tests can be divided into one‐step tests like colonoscopy, which is both diagnostic and therapeutic, and two‐step tests consisting of a stool‐based test or structural examination followed by colonoscopy if positive. Whereas genetic tests in stools and plasma hold promise (Table 2), only the guaiac‐based fecal occult blood test and sigmoidoscopy have been shown to reduce the incidence and/or mortality of CRC in RCTs.^10^

### 6.2 Screening recommendations and performance in PWH

The American College of Gastroenterology updated 2021 guidelines recommend colonoscopy every 10 years and annual fecal immunochemical testing as the primary screening modalities for CRC screening in average‐risk individuals aged 50 to 75 years.^10^ The EACS subscribes to these recommendations.^11^ The USPSTF recommendations (2021) extend the age range for screening to encompass adults aged 45 to 49, with a grade B recommendation.^54^ There is no evidence to date of the benefits of screening for CRC in PWH. Studies assessing the CRC screening rates in PWH compared to the general population have shown inconsistent results.^51^

Table 3 summarizes the harms and costs of the screening methods for CRC.

### 6.3 Implications for management

Despite having a similar frequency to the general population, CRC occurs at younger ages in PWH, which would support earlier initiation of screening. This would be of particular interest in MSM who have been severely immunosuppressed because of the increased risk for rectal squamous cancer.

## 7 SCREENING FOR BREAST CANCER

Breast cancer incidence may be slightly lower in WWH compared to uninfected women.^5^, ^55^ However, they are diagnosed at younger ages and at more advanced stages of disease.^56^, ^57^ In North American and sub‐Saharan African WWH, survival rates were lower than in uninfected women, even after adjusting for classic prognostic risk factors and, in the USA study, for specific cancer treatments.^57^, ^58^

### 7.1 Screening tests

Table 2 shows the screening modalities for breast cancer surveillance.^59^, ^60^ Mammography is the only method that has been shown to reduce breast cancer mortality in several randomized controlled trials, although ultrasound and MRI have demonstrated better sensitivity.^60^

### 7.2 Screening recommendations and performance in WWH

Most scientific society guidelines recommend a biennial mammography for all women aged 50 to 74 years, extendable to some women aged 40 to 50 years based on individual patient characteristics.^59^, ^60^ EACS guidelines recommend mammography every 1–3 years in women aged 50–74 years.^11^ Results about the frequency of breast cancer screening in WWH have ranged from 50% to 80%, with discrepant data when compared to the general population.^61^, ^62^

### 7.3 Implications for management

Both the younger age and more advanced stage at diagnosis in WWH would support earlier initiation of screening, more frequent screening, or adapted screening with more sensitive procedures like ultrasound or MRI, particularly in higher‐risk subgroups. In view of the poorer prognosis of WWH, campaigns addressing the importance of breast cancer screening in WWH are desirable to raise awareness in both physicians and patients.

## 8 SCREENING FOR PROSTATE CANCER

Studies comparing the incidence of prostate cancer in PWH versus the general population have shown discordant results, ranging from lower^63^ to similar incidence rates after adjusting for prostate‐specific antigen (PSA) testing in a study in which Gleason grade was significantly greater in PWH.^64^ Prostate cancer mortality in PWH aged 65 years or older was higher after adjusting for specific cancer treatment.^58^ Along with the lung, the prostate is expected to emerge as the most frequent cancer site in PWH by 2030.^20^

### 8.1 Screening tests

Measurement of PSA blood levels is the standard procedure for prostate cancer screening. The results of a meta‐analysis concluded that PSA testing may have a modest effect in reducing prostate cancer mortality but not overall mortality, and it is associated with biopsy and cancer treatment‐related complications.^65^ However, reported trials had methodological limitations, and there was substantial heterogeneity concerning screening intensity, screening intervals, and PSA biopsy thresholds. Digital rectal examination is no longer recommended as a screening strategy due to the lack of evidence on the benefits.^66^

### 8.2 Screening recommendations and performance in PWH

There are no specific recommendations for prostate cancer screening. The European Association of Urology (EAU) and the USPSTF guidelines advocate shared decision‐making after discussing the potential benefits and harms of screening with the patient. PSA would then be offered to men from 50 years of age or younger men at elevated risk of prostate cancer (EAU),^66^ or to those aged 55 to 69 years (USPSTF).^67^ The EACS guidelines endorse the EAU recommendations.^11^ In a small case–control study conducted in the EuroSIDA cohort, the cutoff used in the general population showed low sensitivity in PWH.^68^

### 8.3 Implications for management

Prostate cancer is expected to be a leading NADC in the next decade as PWH become older. High‐level evidence is needed to determine the usefulness of prostate cancer screening to reduce mortality; alternative screening procedures such as ultrasound or MRI might also be explored to assess their potential advantages over PSA.

**FIGURE 1:**
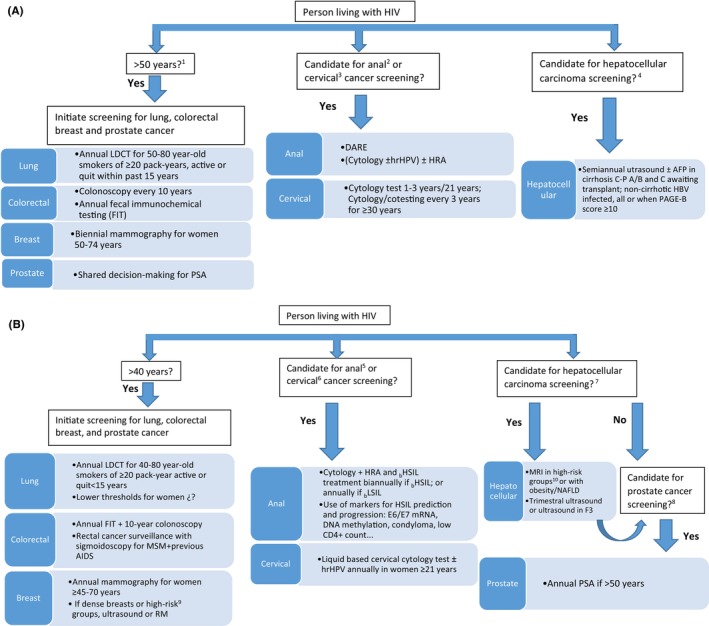
Cancer screening strategies in people living with HIV. (A) Strategies recommended by the European AIDS Clinical Society. (B) Proposed strategies for tailored cancer screening. ^1^≥ 45 years for colorectal cancer screening according to the 2021 US Preventive Services Task Force criteria (grade B). ^2^MSM/transgender women living with HIV and in persons with HPV‐associated dysplasia. ^3^Women ≥21 years. ^4^Cirrhotic persons Child‐Pugh A/B, and C awaiting for LT; non‐cirrhotic HBV infected, all or when PAGE‐B score ≥ 10. ^5^MSM/transgender women living with HIV and in HPV‐associated dysplasia, genital/anal condyloma or anal intercourse. ^6^Women ≥18 y. ^7^Cirrhotic persons Child‐Pugh A/B, and C awaiting for LT; non‐cirrhotic HBV infected, all or when PAGE‐B score ≥ 10. ^8^Men ≥50 years. ^9^High‐risk for breast cancer: known underlying genetic mutation (such as a BRCA1 or BRCA2 gene mutation or other familial breast cancer syndrome) or a history of chest radiation at a young age.^58^ ^10^Higher‐risk persons for hepatocellular carcinoma, those with at least 1% per year risk of developing HCC.^29^ Need to develop algorithms that combine clinical factors and novel biomarkers to identify those patients.

Figure 1 shows the cancer screening strategies recommended by the EACS (Figure 1A) and the proposed strategies for targeted cancer screening in PWH (Figure 1B).

## 9 CONCLUSION

Cancer is the leading cause of mortality in PWH and is expected to account for a growing fraction of deaths as PWH age. Cancer screening has been shown to decrease cancer‐associated mortality in the general population and should be a priority in PWH. In this review, we have compiled the most recent developments in cancer screening and screening performance in PWH, which are currently primarily implemented in well‐resourced settings. This includes an assessment of the associated benefits, harms, and cost‐effectiveness. The article also addresses unmet needs and potential strategies for tailored screening in the HIV population. There is an urgent need to extend the investigation in cancer screening performance to PWH, evaluating whether personalized measures according to individual risk could result in higher efficiency and improve patient outcomes. Randomized intervention studies that assess earlier initiation, more frequent testing, and include the most recent advances in cancer screening are needed to generate high‐level evidence on cancer surveillance tailored to PWH, and to fill existing knowledge gaps.^69^ Nevertheless, owing to the challenges in attaining adequate statistical power to demonstrate a reduction in mortality within in this population, alternative approaches, such as modeling studies, should also be taken into consideration for evidence generation. Meanwhile, strategies for early diagnosis of cancer should be widely implemented in HIV medical care to enhance detection of screenable cancers with the highest excess incidence and mortality. At the same time, further studies also need to be conducted to develop, tailor, and evaluate interventions created to improve screening uptake and adherence in PWH.

## AUTHOR CONTRIBUTIONS

**Mar Masiá:** Conceptualization (equal); data curation (lead); writing – original draft (lead); writing – review and editing (equal). **Ana Gutiérrez Ortiz de la Tabla:** Writing – review and editing (equal). **Félix Gutiérrez:** Conceptualization (equal); funding acquisition (lead); writing – review and editing (equal).

## FUNDING INFORMATION

This work was supported by the RD16/0025/0038 and CB21/13/00011 projects of the National Plan Research + Development + Innovation (R + D + I) and co‐financed by Instituto de Salud Carlos III—Subdirección General de Evaluación y Fondo Europeo de Desarrollo Regional (grants PI16/01740, PI18/01861, CM19/00160, CM20/00066, COV20/00005) and the Conselleria de Innovación, Universidades, Ciencia y Sociedad Digital of the Generalitat Valenciana (grant AICO/2021/205).

## CONFLICT OF INTEREST STATEMENT

The authors declare that they have no conflict of interest.

## Data Availability

Data sharing is not applicable to this article as no new data were created or analyzed in this study.
